# Assessing loss to follow-up in the MObile Technology for Improved Family Planning (MOTIF) randomised controlled trial

**DOI:** 10.1186/s13063-017-2316-6

**Published:** 2017-12-01

**Authors:** Chris Smith, Christopher Jarvis, Caroline Free

**Affiliations:** 1 Department of Population Health, London School of Hygiene and Tropical Medicine, London, WC1E7HT UK; 2 Department of Medical Statistics, London School of Hygiene and Tropical Medicine, London, WC1E7HT UK; 3 Graduate School of Tropical Medicine & Global Health, Nagasaki University, Nagasaki, Japan

**Keywords:** Contraception, Family planning, mHealth, Digital health, Randomised controlled trial, Loss to follow-up, Attrition

## Abstract

**Background:**

Loss to follow-up (LTFU) in clinical trials is an important source of bias that can affect statistical power and generalisability of findings. The aim of this paper is to assess factors associated with LTFU in the MObile Technology for Improved Family Planning (MOTIF) trial in Cambodia and compare how the result might have varied using different analytical methods.

**Methods:**

Follow-up in the MOTIF trial was 86% at 4 months and 66% at 12 months. For the primary analysis, we undertook a complete case analysis, similar to the approach used in similar trials of interventions delivered by mobile phone to increase contraception use. We conducted an exploratory analysis and found that factors associated with LTFU were young age, lower socio-economic status, not planning to use post-abortion contraception, availability of phone credit and not providing additional contact numbers. We then undertook two analyses to estimate the effect of the intervention on the primary outcome at 4 and 12 months for comparison with the complete case analysis. First, we undertook multiple imputation, and second we conducted an analysis treating all participants’ LTFU as non-users of contraception.

**Results:**

Using multiple imputation, we found that the risk ratio was slightly increased at 4 months and slightly decreased at 12 months compared with the complete case analysis. When counting all participants’ LTFU as non-users of contraception, we observed that, compared with the complete case analysis, the risk ratio was slightly decreased at 4 months and slightly increased at 12 months. Despite the changes in the risk ratio, use of the different analytical methods did not result in an effect using the complete case analysis becoming statistically significant or vice versa.

**Conclusion:**

Future studies assessing contraception use might anticipate increased attrition amongst younger participants, those of lower socio-economic status or those who do not provide additional contact details. Attrition could be reduced by collecting as many contact details as possible, by providing incentives and possibly by enhanced counselling to groups at higher risk of LTFU on recruitment. Multiple imputation should be considered in addition to complete case analysis if LTFU not missing at random is expected or observed.

**Trial registration:**

ClinicalTrials.gov, NCT01823861. Registered on 30 March 2013.

## Background

Loss to follow-up (LTFU) in clinical trials is an important source of bias that can affect statistical power and generalisability of findings, and trials with large LTFU can be downgraded in systematic reviews. Systemic differences in LTFU between groups can result in attrition bias [1].

Successful follow-up requires participants to be found and also willing to participate with the data collection procedure [2]. LTFU can be classified as missing at random (MAR) or not missing at random (NMAR), such as increased attrition in younger people. In this situation a complete case analysis, restricted to participants with available follow-up data, may produce a biased estimation of the true effect. A common approach to dealing with missing outcome data is to impute outcomes and treat them as if they were real measurements, known as multiple imputation [3]. The aim of this paper is to assess factors associated with LTFU in the MObile Technology for Improved Family Planning (MOTIF) trial in Cambodia and compare how the result might have varied using different analysis methods.

## Main text

For the MOTIF trial, the primary outcome—self-reported use of an effective contraceptive—was assessed by attempting to contact participants by phone at 4 and 12 months. We used evidence-based methods to try to reduce the chance of LTFU, such as collecting as many possible phone numbers on recruitment, including those of friends or family members, with consent of the participant [4, 5]. At least three attempts were made to contact participants by phone. If it was not a convenient time for the participant to talk, another time would be arranged. At the time of the trial, few participants used email, there was not a functioning postal system in rural areas in Cambodia, and we had limited resources with which to visit participants’ homes. At 4 months we obtained primary outcome data from 431 participants (86%). Amongst the 69 clients LTFU, 6 withdrew, and we were unable to contact 63 participants. At 12 months we obtained primary outcome data from 328 participants (66%). Amongst the 172 clients LTFU, 8 withdrew, and research assistants were unable to contact 164 participants.

For the primary analysis we undertook a complete case analysis, similar to the approach used in similar trials of interventions delivered by mobile phone to increase contraception use [6, 7]. Using a complete case analysis, we found that the intervention increased self-reported use of an effective contraception method at 4 months post-abortion (64% vs. 46%; risk ratio [RR] 1.39, 95% CI 1.17–1.66; *p* < 0.001) but not after 12 months (50% vs. 43%; RR 1.16, 95% CI 0.92–1.47; *p* = 0.208).

### Investigating missing data

Reasons, as documented by the research assistants, for LTFU at 12 months amongst these 164 participants were as follows: 76 (46%) times the phone was switched off; 57 (35%) times there was an automated response that the number was not in use and not yet re-assigned; 16 (10%) times someone else answered the phone; in 10 (6%) cases where the participant had moved abroad; in 3 (2%) cases an automated response indicated that the phone could not receive incoming calls; and in 2 (1%) cases the participant did not want to talk.

We conducted an exploratory analysis to identify predictors of LTFU from the variables collected at trial recruitment to assess whether data were MAR or NMAR. To maximise statistical power, binary variables were created from some of the categorical variables. First we undertook univariable analysis to examine the crude association between each baseline variable and LTFU, expressed as an OR with a 95% CI. In the univariable analysis, age < 25 years, lower socio-economic status (SES; i.e., no access to motorised transport), not planning to use post-abortion contraception and not providing an additional friend/family contact number were associated with LTFU at 4 months (Table 1). Age < 25 years, lower SES and usually or sometimes having phone credit were associated with LTFU at 12 months (Table 2). There was no evidence of differential LTFU between those assigned to the intervention or control arm at 4 or 12 months.

**Table 1:** Predictors of missing data at 4 months

	Followed up (*n* = 431)	LTFU (*n* = 69)	Crude OR	*p* Value	Adjusted OR^a^	*p* Value
Age group						
Age > 25 years (r)	306 (89%)	37 (11%)	1.00			
Age < 25 years	125 (80%)	32 (20%)	2.12 (1.26–3.55)	0.004	2.31 (1.36–3.92)	0.002
Residence						
Rural (r)	279 (87%)	42 (13%)	1.00			
Urban	152 (85%)	27 (15%)	1.18 (0.70–1.99)	0.535	1.11 (0.65–1.90)	0.699
Socio-economic status						
Access to motorised transport (r)	381 (88%)	54 (12%)	1.00			
No access to motorised transport	50 (77%)	15 (23%)	2.12 (1.11–4.03)	0.022	2.44 (1.26–4.72)	0.008
Education						
Secondary or above (r)	263 (87%)	41 (13%)	1.00			
None or primary	168 (86%)	28 (14%)	1.07 (0.64–1.79)	0.800	1.14 (0.64–2.02)	0.665
Marital status						
Married/living together (r)	403 (87%)	61 (13%)	1.00			
Never married or living together	23 (79%)	6 (21%)	1.72 (0.67–4.40)	0.255	1.32 (0.51–3.43)	0.573
Divorced/separated	5 (71%)	2 (29%)	2.64 (0.50–13.92)	0.252	1.31 (0.22–7.75)	0.769
No. of living children						
1 or more (r)	312 (88%)	41 (12%)	1.00			
0	119 (81%)	28 (19%)	1.79 (1.06–3.03)	0.030	1.31 (0.71–2.42)	0.382
No. of previous abortions						
0 (r)	254 (85%)	45 (15%)	1.00			
1 or more	177 (88%)	24 (12%)	0.77 (0.45–1.30)	0.324	0.91 (0.52–1.57)	0.724
Contraception decision-making						
Joint decision (r)	257 (89%)	33 (11%)	1.00			
Mainly participant	79 (82%)	17 (18%)	1.68 (0.89–3.17)	0.112	1.76 (0.91–3.37)	0.091
Mainly husband/partner	66 (85%)	12 (15%)	1.42 (0.69–2.89)	0.340	1.58 (0.76–3.26)	0.220
Mobile phone access						
Never shares (r)	225 (87%)	34 (13%)	1.00			
Shares	206 (85%)	35 (15%)	1.12 (0.68–1.87)	0.651	1.21 (0.72–2.03)	0.474
Disclosure of abortion to others						
Yes (r)	174 (85%)	30 (15%)	1.00			
No	32 (86%)	5 (14%)	0.91 (0.33–2.51)	0.850	1.10 (0.39–3.16)	0.853
PAFP intentions						
Yes (r)	166 (89%)	21 (11%)	1.00			
No	31 (74%)	11 (26%)	2.80 (1.23–6.39)	0.014	2.34 (1.00–5.46)	0.050
Undecided	234 (86%)	37 (14%)	1.25 (0.71–2.21)	0.444	1.13 (0.63–2.01)	0.689
Fertility plans						
No more/none (r)	123 (88%)	16 (12%)	1.00			
Have a/another child	261 (85%)	47 (15%)	1.38 (0.75–2.54)	0.293	1.00 (0.50–1.96)	0.990
Undecided	47 (89%)	6 (11%)	0.98 (0.36–2.66)	0.971	0.88 (0.32–2.45)	0.810
Abortion method						
Medical (r)	178 (86%)	29 (14%)	1.00			
Surgical	253 (86%)	40 (14%)	0.97 (0.58–1.62)	0.909	1.00 (0.59–1.70)	0.990
Phone credit						
Always (r)	158 (88%)	21 (12%)	1.00			
Usually	102 (80%)	26 (20%)	1.92 (1.02–3.59)	0.042	1.71 (0.90–3.24)	0.101
Sometimes	171 (89%)	22 (11%)	0.97 (0.51–1.83)	0.920	0.88 (0.45–1.71)	0.705
Friend/family contact provided						
Yes (r)	338 (88%)	44 (12%)	1.00			
No	93 (79%)	25 (21%)	2.07 (1.20–3.55)	0.009	2.40 (1.37–4.21)	0.002
No. of phone numbers provided						
Two or more (r)	128 (89%)	16 (11%)	1.00			
One	303 (85%)	53 (15%)	1.40 (0.77–2.54)	0.269	1.42 (0.77–2.59)	0.261
Randomisation						
Intervention (r)	211 (85%)	38 (15%)	1.00			
Control	220 (88%)	31 (12%)	0.78 (0.47–1.30)	0.346	0.81 (0.48–1.37)	0.431

*LTFU* Loss to follow-up, *PAFP* Post-abortion family planning, (r): Reference category

^a^Adjusted for age and SES. *P* values less than 0.05 were regarded as significant. Analyses were undertaken using STATA 13.1 software (StataCorp, College Station, TX, USA)

**Table 2:** Predictors of missing data at 12 months

	Followed up (*n* = 328)	LTFU (*n* = 172)	Crude OR	*p* Value	Adjusted OR^a^	*p* Value
Age group						
Age > 25 years (r)	235 (69%)	108 (31%)	1.00			
Age < 25 years	93 (59%)	64 (41%)	1.50 (1.01–2.21)	0.043	1.59 (1.07–2.36)	0.023
Residence						
Rural (r)	212 (66%)	109 (34%)	1.00			
Urban	116 (65%)	63 (35%)	1.06 (0.72–1.55)	0.780	1.04 (0.70–1.54)	0.848
Socio-economic status						
Access to motorised transport (r)	294 (68%)	141 (32%)	1.00			
No access to motorised transport	34 (52%)	31 (48%)	1.90 (1.12–3.22)	0.017	2.04 (1.19–3.47)	0.009
Education						
Secondary or above (r)	209 (69%)	95 (31%)	1.00			
None or primary	119 (61%)	77 (39%)	1.42 (0.98–2.07)	0.065	1.48 (0.98–2.23)	0.060
Marital status						
Married/living together (r)	309 (67%)	155 (33%)	1.00			
Never married or living together	16 (55%)	13 (45%)	1.62 (0.76–3.45)	0.212	1.38 (0.64–2.99)	0.416
Divorced/separated	3 (43%)	4 (57%)	2.66 (0.59–12.02)	0.204	1.51 (0.31–7.41)	0.613
No. of living children						
1 or more (r)	241 (68%)	112 (32%)	1.00			
0	87 (59%)	60 (41%)	1.48 (1.00–2.21)	0.052	1.29 (0.81–2.04)	0.280
No. of previous abortions						
0 (r)	201 (67%)	98 (33%)	1.00			
1 or more	127 (63%)	74 (37%)	1.20 (0.82–1.74)	0.351	1.34 (0.91–1.98)	0.136
Contraception decision-making						
Joint decision (r)	193 (67%)	97 (33%)	1.00			
Mainly participant	63 (66%)	33 (34%)	1.04 (0.64–1.70)	0.868	1.06 (0.65–1.74)	0.812
Mainly husband/partner	53 (68%)	25 (32%)	0.94 (0.55–1.60)	0.816	0.99 (0.57–1.69)	0.959
Mobile phone access						
Never shares (r)	175 (68%)	84 (32%)	1.00			
Shares	153 (63%)	88 (37%)	1.20 (0.83–1.73)	0.337	1.25 (0.86–1.81)	0.252
Disclosure of abortion to others						
Yes (r)	134 (66%)	70 (34%)	1.00			
No	19 (51%)	18 (49%)	1.81 (0.89–3.68)	0.099	1.97 (0.96–4.05)	0.066
PAFP intentions						
Undecided (r)	181 (67%)	90 (33%)	1.00			
Yes	120 (64%)	67 (36%)	1.12 (0.76–1.66)	0.562	1.20 (0.81–1.79)	0.360
No	27 (64%)	15 (36%)	1.12 (0.57–2.21)	0.749	1.05 (0.53–2.10)	0.886
Fertility plans						
No more/none (r)	90 (65%)	49 (35%)	1.00			
Have a/another child	197 (64%)	111 (36%)	1.03 (0.68–1.57)	0.872	0.87 (0.55–1.38)	0.561
Undecided	41 (77%)	12 (23%)	0.54 (0.26–1.12)	0.096	0.51 (0.24–1.08)	0.080
Abortion method						
Medical (r)	138 (67%)	69 (33%)	1.00			
Surgical	190 (65%)	103 (35%)	1.08 (0.74–1.58)	0.673	1.09 (0.74–1.60)	0.651
Phone credit						
Always (r)	132 (74%)	47 (26%)	1.00			
Usually	75 (59%)	53 (41%)	1.98 (1.22–3.22)	0.006	1.85 (1.13–3.02)	0.014
Sometimes	121 (63%)	72 (37%)	1.67 (1.07–2.60)	0.023	1.59 (1.01–2.51)	0.046
Friend/family contact provided						
Yes (r)	250 (65%)	132 (35%)	1.00			
No	78 (66%)	40 (34%)	0.97 (0.63–1.50)	0.896	1.03 (0.66–1.60)	0.897
No. of phone numbers provided						
Two or more (r)	99 (69%)	45 (31%)	1.00			
One	229 (64%)	127 (36%)	1.22 (0.81–1.85)	0.346	1.22 (0.80–1.85)	0.357
Randomisation						
Intervention (r)	169 (68%)	80 (32%)	1.00			
Control	159 (63%)	92 (37%)	1.22 (0.84–1.77)	0.287	1.25 (0.86–1.81)	0.253

*LTFU* Loss to follow-up, *PAFP* Post-abortion family planning, (r): Reference category

^a^Adjusted for age and SES. *P* values less than 0.05 were regarded as significant. Analyses were undertaken using STATA 13.1 software.

We then undertook multivariable analysis to explore the association between variables and LTFU, controlling for confounding, using logistic regression analysis. Age and SES are associated with contraception use and may be associated with seeking LTFU [8, 9]. We therefore considered these variables as confounders in the analysis. In the multivariable analysis, age < 25 years, lower SES, not planning to use post-abortion contraception and not providing an additional friend/family contact number remained as predictors of LTFU at 4 months; LTFU was significantly increased amongst women aged < 25 years compared with those aged > 25 years (OR 2.31; *p* = 0.002), amongst women without access to motorised transport compared with women with access to motorised transport (adjusted OR 2.44; *p* = 0.008), amongst women not planning to use post-abortion contraception compared with women planning to use it (OR 2.34; *p* = 0.05), and amongst women who did not provide additional contact numbers compared with those who did (OR 2.40; *p* = 0.002) (Table 1).

At 12 months, age < 25 years, lower SES, and phone credit remained as predictors of LTFU; LTFU was significantly increased amongst women aged < 25 years compared with those aged > 25 years (OR 1.59; *p* = 0.023), amongst women without access to motorised transport compared with women with access to motorised transport (OR 2.04; *p* = 0.009), and amongst women who usually (OR 1.85; *p* = 0.014) or sometimes (OR 1.59; *p* = 0.046) had phone credit compared with women who always had phone credit (Table 2).

We then undertook two different analyses to compare with the complete case analysis (Table 3). First, we undertook multiple imputation to estimate the effect of the intervention on the primary outcome at 4 and 12 months. This involved replacing each missing primary outcome value with a plausible imputation based on the characteristics of missing values observed [10]. Because LTFU was NMAR, variables associated with LTFU were used in the multiple imputation model. Thus, logistic regression with the predictor variables age group, SES, post-abortion family planning intentions and whether an additional friend or family contact was provided was used for univariate imputation of the outcome at 4 months and the predictor variables age group, SES and phone credit for imputation of the outcome at 12 months. Multiple imputation was conducted separately for the outcomes at 4 and 12 months with 100 imputed datasets created for each outcome, respectively. Using multiple imputation, the intervention also showed an effect at 4 months (63% vs. 45%; RR 1.42, 95% CI 1.19–1.69; *p* = 0.001) but not at 12 months (48% vs. 43%; RR 1.12, 95% CI 0.89–1.40; *p* = 0.349). Second, we conducted sensitivity analyses treating all participants LTFU as non-users of contraception (extreme case scenario) to estimate the effect of the intervention on the primary outcome at 4 and 12 months [11]. In this analysis self-reported effective contraceptive use also remained significantly increased when we considered participants LTFU as non-users at 4 months (54% vs. 40%; RR 1.35, 95% CI 1.12–1.63; *p* = 0.002) but not at 12 months (34% vs. 27%; RR 1.25, 95% CI 0.95–1.63; *p* = 0.106).

**Table 3:** Effect measure variation according to analytical method used

	Intervention group, *n* (%)	Control group, *n* (%)	RR (95% CI)	*p* Value	Intervention group, *n* (%)	Control group, *n* (%)	RR (95% CI)	*p* Value
	4 months				12 months			
Complete case analysis	135/211 (64%)	101/220 (46%)	1.39 (1.17–1.66)	<0.001	84/169 (50%)	68/159 (43%)	1.16 (0.92–1.47)	0.208
Counting participants LTFU as non-users	135/249 (54%)	101/251 (40%)	1.35 (1.12–1.63)	0.002	84/249 (34%)	68/251 (27%)	1.25 (0.95–1.63)	0.106
Multiple imputation	158/249 (63%)	114/251 (45%)	1.42 (1.19–1.69)	< 0.001	120/249 (48%)	108/251 (43%)	1.12 (0.89–1.40)	0.349

*LTFU* Loss to follow-up, *RR* Risk Ratio

Table 3 shows how the effect measure varies according to the analytical method used. Using multiple imputation, we found that the RR was slightly increased at 4 months and slightly decreased at 12 months compared with the complete case analysis. When counting all participants LTFU as non-users of contraception, we observed that, compared with the complete case analysis, the RR was slightly decreased at 4 months and slightly increased at 12 months. Despite the changes in the RR, use of the different analytical methods did not result in an effect becoming statistically significant or vice versa.

## Conclusion

Factors associated with LTFU in the MOTIF trial were young age, lower SES, not planning to use post-abortion contraception, phone credit and not providing additional contact numbers. Reasons for LTFU amongst younger women might be that they changed phone numbers (could not be found) or perhaps the potential stigma of contraceptive use in young people (no longer wanting to participate in the trial). Younger participants were also found to be at increased risk of LTFU in a head injury trial [2]. LTFU amongst women of lower SES might be due to changing phone numbers or no longer having access to a phone. Women not planning to use contraception might have been less motivated to participate in the trial follow-up. The finding that LTFU was increased in those not providing multiple contacts is consistent with existing evidence [4]. Although the RR showed slight variation using multiple imputation and counting those LTFU as non-users, there was no significant change in the principal findings.

Whilst this study provides some insights into factors associated with LTFU in this trial, it may have limited generalisability to other studies. This study had a relatively small sample size and was an exploratory rather than a pre-specified analysis.

Future studies assessing contraception use might anticipate increased attrition amongst younger participants and those of lower SES or who do not provide additional contact details. Attrition could be reduced by collecting as many contact details as possible, providing incentives, and possibly providing enhanced counselling to groups at higher risk of LTFU on recruitment [4]. Multiple imputation should be considered in addition to complete case analysis if LTFU NMAR is expected or observed.

## Abbreviations

LTFU: Loss to follow-up
MAR: Missing at random
MOTIF: MObile Technology for Improved Family Planning
NMAR: Not missing at random
PAFP: Post-abortion family planning
RR: Risk Ratio
SES: Socio-economic status
